# Surveying people with spinal cord injuries in Brazil to ascertain research priorities

**DOI:** 10.1038/s41598-022-26733-7

**Published:** 2023-01-12

**Authors:** Fabiana Faleiros, Deyse Cardoso de Oliveira Braga, Soraia Dornelles Schoeller, Sílvia Helena Henriques, Naira Beatriz Favoretto Cunha, Lorena Gomes Neves Videira, Adriana Cordeiro Leandro da Silva Grillo

**Affiliations:** 1 Ribeirão Preto College of Nursing, University of Sao Paulo, Ribeirão Preto, Brazil; 2 Nursing Department, University of Santa Catarina, Florianópolis, Brazil

**Keywords:** Neuroscience, Health care, Medical research, Neurology

## Abstract

Scientists are concerned that the research they conduct accurately portrays the needs of people living with spinal cord injuries (SCI). As such, this study set out to investigate the main problems faced by people with SCI and their expectations for research. This quantitative, exploratory, analytical, and cross-sectional study was carried out online, with a non-probabilistic sample of 618 Brazilian adults with SCI who had registered voluntarily to participate in the research of the Neurorehab group. The virtual questionnaire consisted of 22 questions based on ISCOS Data Sets. The majority (68.9%) of participants were male, with higher education or a post-graduate qualification (49.5%). Most injuries had experienced traumatic injuries (78.5%) and 58.7% were paraplegic. The mean age was 38.04 years (SD = 9.85). The main difficulties faced after SCI were locomotion/accessibility (70.9%), neurogenic bladder (68.8%), neurogenic bowel (48.2%), and sexuality (36.1%). The highest demand was for experimental studies on stem cells (22.5%), rehabilitation (14.2%), and cures (13.9%). Most (84.3%) of those who reported sexuality problems after SCI were men (p = 0.013). The findings obtained empower people with SCI by enabling them to influence the agenda of scientific research based on their expectations and difficulties. This survey will also aid organizations to engage stakeholders to implement a comprehensive SCI management program.

## Introduction

Spinal cord injury (SCI) is associated with changes in health and functionality. This can lead to functional compromise and social restrictions, as a result of health problems or accessibility difficulties in the environment in which they live^1,2^. The quality of life of people living with SCI is influenced by the impaired functions of motor, autonomic nerve system, intestinal, bladder, and sexual, which have a substantial impact on the well-being of these individuals, in addition to other social aspects of life, such as relationships, emotional factors, and access to the labor market^1–3^.

The actions of the World Health Organization (WHO) and recent research have elucidated the value of rehabilitation as a key health strategy of the twenty-first century^4^. Particularly considering the aim of rehabilitation to "optimize functioning and reduce disability", according to the International Classification of Functioning, Disability and Health (ICF), as a starting point^4^. Autonomy and social participation are the ultimate goals of rehabilitation. Autonomy is associated with the capacity of people to make decisions about their own lives, constructing and generating strategies of self-care and self-control^5^. Therefore, it makes sense for people with SCI, including from an ethical point of view, to actively participate in the course of research on SCI and rehabilitation, identifying problems and strategies that could work for them, and making them essential partners for researchers in this area. This alludes to the celebrated Oliver, advocate of the social model of disability, who defended the idea that much of the inconvenience and difficulty of living with a disability is not an inherent feature of the disability itself, but a failure of society to adapt to the needs of disabled people. Also coined the term "Emancipatory disability studies" by which meant that researchers must not be "parasites" but instead serve the interests of disabled people^6^.

Previous experiences around the world have shown that clinical practices obtained through the inclusion of users in the process improve care and advance the base of knowledge in the ***field^7–9^. One example is the Public and Patient Involvement program in the United Kingdom, which helps to identify relevant research topics, improve the appropriateness of research results, and ensure that research findings are shared with society^7^. Engaging these stakeholders actively in discussions on patient involvement early in the agenda‐setting process and keeping them informed about the outcomes help to build enduring partnerships^8,9^.

Recent international publications have indicated that the opinions, values, expectations, and fears of individuals with SCI may be a valuable resource for scientists and clinicians in this field of research, but are frequently overlooked^8–10^. In Brazil, there is a knowledge gap in this sense, international multicentric research is mostly related to the perception of quality of life of people living with SCI^11^.

The present study aims to ensure that the patient’s opinion is considered in future scientific studies and in the development of clinical trials, also in Brazil. In light of the above, and to support the participation of people with SCI in the scientific process, this study aims to identify and analyze the main problems/difficulties faced after spinal cord injury and the topics suggested by participants with SCI for future research. It is hoped that this study will guide the forthcoming investigations of Brazilian research groups working with the rehabilitation of people with SCI.

## Method

This is a quantitative, exploratory, analytical, and cross-sectional study. The research project was approved by Universidade de São Paulo’s Comitê de Ética e Pesquisa-CEP (Research Ethics Committee), as per the Health Ministry’s Conselho Nacional de Ética em Pesquisa (National Council for Ethics in Research) Resolution 466/12, which addresses ethics in research with human beings under protocol no. CAAE: 07814219.6.0000.5393. All research was performed in accordance with relevant guidelines/regulations, and informed consent was obtained from all participants.

Data for this study were collected online from participants across Brazil. A non-probabilistic sample was composed of 618 Brazilian adults with spinal cord injury and internet access who had registered voluntarily to participate in research on SCI and were on the database of the Núcleo de Pesquisa e Atenção em Reabilitação Neuropsicomotora—NEUROREHAB (Nucleus for Research and Care in Neuropsychomotor Rehabilitation) research group. Participants who did not completely answer all the questions on the questionnaire were excluded. The online form was started by 966 people, 618 of whom completed it and met the eligibility criteria. The response rate was 64%, with an average form completion time of 11 min.

An online form was used for voluntary registration, which was created on the Survey Monkey^®^ virtual platform and validated in a previous study. The virtual form, which can be visualized at https://pt.surveymonkey.com/r/cadastroLM_NEUROREHAB_publicacoes was widely promoted on social media, with posts directed at groups with the target audience of the research and digital influencers in the circle of people with disabilities. The questionnaire developed to characterize the profile of the participants consisted of 22 questions based on ISCOS Data Sets^11^. Question 20 on the form proposed that the participants offer opinions on the topics of research they would like to see conducted in the area of SCI. However, during data collection, it was noted that many of the participants were focusing only on experimental research (stem cells) when answering this question. Therefore, an optional question was added to the form in which the participants were asked about the main difficulties/problems they faced after SCI.

The data collected through the SurveyMonkey^®^ platform were transferred directly to the Statistical Package of Social Science version 22.0 (IBM, 2013) and R version 3.3.0 (R CORE TEAM, 2016) statistical programs.

Two meetings were held among the specialists to organize the collected data based on the similarity of the cited topics, as follows: a meeting to define the categories; individual work to analyze said categories; and a meeting to reach a consensus on the categories and adjust the nomenclature.

Descriptive statistics were employed to analyze the data through absolute frequencies and calculation of the mean, median, percentage, standard deviation, minimum and maximum. Contingency tables and Pearson’s Chi-Squared test were used for the categorical variables. The Mann–Whitney test was used for comparison between the problems/difficulties after SCI (categorical variable) and age and time since SCI (numerical variables). The significance level was 0.05.

The datasets generated and analysed during the current study are not publicly available due have been collected ensuring their anonymity, but are available from the corresponding author on reasonable request.

## Results

### Participants

The sample was composed of 618 participants, 426 (68.9%) of whom were male and 192 (31.1%) female. The age of the participants ranged from 18 to 70 years, with a mean of 38.04 years (± 9.85).

Regarding region of origin, 387 (62.6%) participants were from the Southeast of Brazil, 105 (17%) from the South, 65 (10.5%) from the Northeast, 47 (7.6%) from the Central-West, and 17 (2.3%) from the North. Regarding level of education, 306 participants (49.5%) reported complete or incomplete higher education, 232 (37.5%) declared complete or incomplete secondary education, 78 (12.6%) had complete or incomplete primary education, and 0.3% did not respond.

In relation to the level of the spinal cord injury, 301 (48.7%) participants reported an injury at the thoracic level, 232 (37.5%) at the cervical level, 53 (8.6%) at the lumbar level, and 32 (5.2%) at the sacral level. The most SCI causes were traumatic 485 (78.5%). The mean time since the spinal cord injury was 9.3 years (± 7.9). The mean age at the time of SCI was 28.14 (SD = 10.24).

### Research topics cited by the participants

A total of 496 (80.3%) participants expressed the research topics that they would like to see addressed in forthcoming studies on SCI and rehabilitation. The responses were organized into 12 categories selected by three specialists with experience in the area of rehabilitation. The most cited were stem cell research 144 (23%), rehabilitation 91 (15%), and cure 89 (14%), among others (Table 1).

**Table 1:** Sample distribution according to the main research themes highlighted by the participants (n = 496), Brazil, 2018.

Research themes	Frequency (n)	Percentage
Stem cells	144	23
Rehabilitation	91	15
Cure	89	14
Bladder	60	9
Treatments	54	2
Tecnology	42	7
Bowel	38	6
Pain	34	6
Sexuality	15	2
Psychological aspects	14	2
Others	60	10
No reply	122	20

It should be highlighted that each participant could indicate more than one research topic, resulting in a total number of responses of 818 from the 496 individuals that answered the question.

The others category (10%) (Table 1) covers topics that were cited by the participants but were not included among the other topics, as they appeared less frequently. This option included wounds/skin (9), myelitis (11), spasms (11), and other responses that did not fit into any of the other categories (29).

### Main difficulties faced after SCI listed by the participants

**Figure 1:**
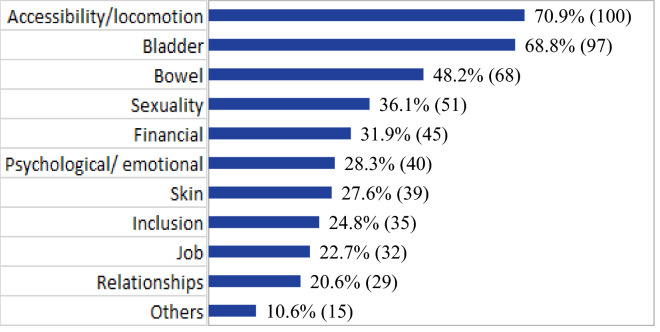
Distribution of the main difficulties/problems after spinal cord injury (n = 141), Brazil, 2018.

As previously described, not all the research participants had the opportunity to answer the question on difficulties after SCI since it was added to the form after data collection had begun. Thus, after inclusion, 172 participants viewed the question and 141 (81.2%) responded. The responses to the main problems and difficulties faced after spinal cord injury included accessibility/locomotion 100 (70.9%), bladder function 97 (68.8%), bowel function 68 (48.2%), sexuality 51 (36.1%) and financial problems 45 (31.9%). The others category (10.6%) mainly included difficulties with pain and balance (Fig. 1).

Statistical tests were carried out to investigate the association between the difficulties experienced after SCI and the variables age and time since SCI (Table 2) and origin and gender (Table 3).

**Table 2:** Presentation of test results between “difficulties after SCI” and the variables “age” and “time of spinal cord injury”, Brazil, 2018.

Difficulties after SCI	Age	Time after injury
	p value	
Accessibility/locomotion 70.9%(100)	**0.022**	0.351
Bladder 68.8% (97)	0.140	0.333
Bowel 48.2% (68)	0.558	0.490
Sexuality 36.1% (51)	0.147	0.936
Financial 31.9% (45)	0.394	0.794
Emotional aspects 28.3% (40)	0.355	**0.001**
Skin 27.6% (39)	0.287	0.757
Inclusion 24.8% (35)	0.723	0.452
Job 22.7% (32)	0.460	0.139
Relationship 20.6% (29)	0.628	0.893

*Teste Mann–Whitney.

Significant values are in [bold].

**Table 3:** Presentation of test results between “difficulties after SCI” and the variables “origin” and “sex”, Brazil, 2018.

Difficulties after SCI	Gender
	p value
Accessibility/locomotion 70.9%(100)	0.625
Bladder 68.8% (97)	0.974
Bowel 48.2% (68)	0.089
Sexuality 36.1% (51)	**0.013**
Financial 31.9% (45)	0.508
Emotional aspects 28.3% (40)	0.578
Skin 27.6% (39)	0.067
Inclusion 24.8% (35)	0.145
Job 22.7% (32)	0.446
Relationship 20.6% (29)	0.409

*Pearson's Chi-Square Test.

Significant values are in [bold].

Table 2 shows statistically significant differences in the correlations between the variables age and accessibility and time since SCI and emotional aspect. The mean age of the people that reported problems/difficulties with accessibility/locomotion after SCI (40.51 years, ± 10.63) was greater than that of the participants that did not report problems (37.40 years, ± 9.34). However, time since injury (6.05 years, ± 7.30) was lower for participants that reported emotional problems/difficulties after SCI than for those that did not report this difficulty (9.55 years, ± 7.90).

Table 3 shows a statistically significant difference in the association between the sexuality variable and the gender variable (p = 0.013, Pearson’s Chi-Squared test); of the 51 people that reported difficulties with Sexuality, the majority (84.3%) were male.

## Discussion

The sample was composed predominantly of young men, with higher education or a post-graduate qualification, traumatic spinal cord injuries and were paraplegic. The main difficulties faced after SCI were locomotion/accessibility, neurogenic bladder, neurogenic bowel and sexuality. The highest demand was for experimental studies on stem cells, rehabilitation and curative therapies.

The size of the sample and the fact that the participants came from all regions of Brazil demonstrates that the study population represented people with spinal cord injury from all over the country. The greater prevalence of males, with a mean age of 38 years, corroborates previous findings in national and international studies. This is attributable the greater involvement of young men in high-risk situations including physical violence, road traffic accidents, or accidents at work^12–14^.

A large proportion of the sample (49.5%) had a high level of education, which contradicts national data on the educational level of people with a physical disability in Brazil^15^. However, as participation and data collection in this study relied on the internet, these results corroborate other studies in which the number of people that use new information and communication technologies, such as the internet, tends to increase with a higher level of education^14,16^.

The largest number of spinal cord injuries were at thoracic level, followed by the cervical, lumbar, and sacral levels, indicating a sample composed mostly of individuals with paraplegia, according to the ASIA criteria^11–14^. Injuries that are comparable in level and extent can produce different magnitudes of functional compromise and subsequent recuperation; however, when the injury is higher up, the level of the injury may influence the level of physical dependence^2^.

The mean time since injury observed in this study was around 9 years, which confirms that advances in the area of rehabilitation, treatment, and care have increased the survival and longevity of individuals with SCI^14,17^. When this data is cross-referenced with emotional factors, it can be observed that the participants who reported emotional problems/difficulties were those who had lived with an injury for a shorter period. According to data in the literature, the prevalence of depressive symptoms after SCI is substantially higher than in the general population^18^ and individuals with a more recent SCI have a higher rate of psychological difficulties^19^. It should be emphasized that SCI involves an intense rupture in relation to the patient’s previous life and that the more recent the injury, the greater the impact and insecurity regarding future possibilities of autonomy and participation. At the same time, although the aging process leads to certain limitations, age may be a protective factor than strengthens the psychological capacity of people with SCI to cope with their situation.

This study identified the topics for which people with SCI would like to see more research. Most of the participants related this question to experimental research with stem cells and to curing the disease, probably due to the image that the population has of scientists in experimental laboratories conducting research. In addition, the findings indicate a desire among people with SCI for complete reestablishment of their health, as the most frequent responses requested research aimed at spinal cord regeneration (stem cells), rehabilitation, cure, treatment, and technologies. The topic of cure has been frequently mentioned in previous studies in which research is requested by the participants^20,21^, which corroborates the results of the present study. The high frequency of the term stem cells may be associated with the wide dissemination of research on this topic in the Brazilian media, representing renewed hope of walking again and cure for people with SCI. The prioritized topics are intimately entwined, as stem cells may bring what the participants consider a cure for SCI and for the problems resulting from the injury related to bladder function, bowel function, motor skills, etc.

We realize that the Brazilian population does not have knowledge about the breadth of scientific research and evidence-based practice. A question of the form proposed that the participants offer opinions on the topics of research they would like to see conducted in the area of SCI. However, during data collection, it was noted that many of the participants were focusing only on experimental research (stem cells) when answering this question. Therefore, an optional question was added to the form in which the participants were asked about the main difficulties/problems they faced after SCI.In view of this, we had to change the conduct of the research to be able to identify the real problems after SCI, in order to turn them into research problems in the future. It became clear that we need to promote the scientific education of the population, so that we can have an effective participation of people with disabilities in our research.

The biggest problems/difficulties after spinal cord injury mentioned by the participants were loss of motor function associated with the SCI (locomotion and accessibility), followed by neurogenic bladder and bowel (incontinence), sexuality, and financial, psychological, and emotional aspects. Skin problems, inclusion, employment, and relationships were reported less frequently. In other words, the people with SCI priorities accessibility but wish to have some research about recovery and stem cell therapy.

The international literature provides information in respect to people living with SCI’s perception of the domains that most affect their satisfaction with life, such as the ability to self-care^11^. Other studies in Brazil also mention the difficulty with locomotion, followed by dissatisfaction with the employment situation, as factors that negatively affect the quality of life of this population^22^.

In Brazil, accessibility is guaranteed by legal mechanisms such as Law no. 13.146/2015-Lei Brasileira de Inclusão da Pessoa com Deficiência (Brazilian Law for the Inclusion of People with Disabilities) and Law no. 10***.098/2000, which establishes general norms and basic criteria for the promotion of accessibility for this population. Despite the legislation supporting the rights of people with disabilities, data from the demographic census of 2010, conducted by the Instituto Brasileiro de Geografia e Estatística (IBGE—Brazilian Institute of Geography and Statistics), demonstrates that the vast majority of the approximately 45.6 million people in Brazil with some kind of disability get by without access to healthcare, education, or rehabilitation^23^. This also corroborates the result that most of the respondents (70.9%) reported the issue of accessibility as a difficulty.

When cross-referencing the variables of accessibility/locomotion with the origin of the participants, the North (Amazon rainforest region) region provided the highest proportion of individuals who mentioned difficulty with accessibility after SCI. We associated this with the fact that the North is considered the region with the poorest development structure and the lowest availability of resources. Moreover, it is geographically isolated in relation to the more developed regions of the country, with river transport being commonly used, and it suffers from high rates of poverty^15,16^.

Difficulties with access to employment and the consequent financial repercussion after SCI were raised as problems by the study participants. When it is not possible to return to work or to become qualified in order to increase chances of employment, this triggers a process through which more people become dependent on welfare payments and disability benefits. This increases the burden on the welfare system and results in little economic contribution to the country, given that most people with SCI are young and at an economically active age. In addition, the main rehabilitation centers for people with SCI in Brazil target capacity-building for self-care and daily living activities; interventions that prepare the person with SCI to return to the labor market are scarce, including, for example, the adaptations needed to conduct work activities in a wheelchair, for example^5,14^.

Also regarding locomotion, the results of this study show an association between age and difficulty with locomotion/accessibility. A previous study showed that the autonomy and participation of elderly people living with SCI is hampered by accessibility difficulties since aging is associated with loss of muscle mass and strength, which can hinder tasks such as maneuvering the wheelchair or overcoming obstacles^24^. The physical barriers and the difficulties of urban mobility promote the confinement of these people to their homes, generating feelings of inferiority and, consequently, social exclusion. This was also shown in the present study since social inclusion is a problem for 24.8% of the participants.

Urinary and intestinal excretions, represented by neurogenic bladder and bowel, appear in both the suggestions for research and in the list of main problems associated with SCI, in second and third position, respectively. Neurogenic bladder and bowel management, with its complications related to incontinence, constipation, urinary tract infections, and intermittent urinary catheterization, is widely recognized and researched, corroborating the findings of this study^1,25^. Changes to bladder and bowel functions negatively affect the quality of life and social participation of people with SCI. In addition to being related to high rates of morbidity and mortality, due to renal deterioration, there are also negative effects related to inclusion in social, educational, labor, and sexual activities^26^.

Sexuality was the fourth most reported problem by the study participants and appeared on the list of research suggestions. Most (84.3%) of the participants that reported problems with sexuality were male. In addition to the possibility of erectile dysfunction and alterations to male ejaculation, people of both sexes with SCI have reported reduced sexual activity and satisfaction in previous studies. This can be attributed to hindered mobility, urinary and fecal incontinence, sensitivity, pain, embarrassment towards their own body, low self-esteem, difficulty reaching orgasm, and difficulty maintaining lubrification^27,28^. Furthermore, this result shows that interventions in sexual dysfunction after SCI should be developed, as international studies have found that sexual dysfunction exhibited the highest self-reported level of severity, but with the lowest treatment rate^29^.

One of the problems after SCI reported by participants concerns the integrity of compromised skin, which occurs as a result of changes in sensitivity and mobility, making areas with prominent bones more susceptible to ischemic phenomena. Currently, nursing interventions aimed at preventing skin lesions in patients with SCI are being implemented with greater frequency by rehabilitation nurses by taking precautions to prevent lesions resulting from pressure, which often limit the participation of people in rehabilitation programs^30^.

Recently, a multinational cross-sectional survey, described the prevalence and correlates of self-reported physical health conditions in persons with spinal cord injury (SCI) from 21 countries, representing all the 6 World Health Organization regions, with 11,058 participants in the International SCI Community Survey (InSCI)^29^. The survey, based on the International Classification of Functioning, Disability and Health (ICF) Core Sets for SCI identified that 95.8% of the participants reported having experienced 1 or more health problems secondary to SCI. Having pain was the most prevalent problem (77.3%), followed by spasticity/muscle spasms (73.5%), sexual dysfunction (71.3%), bowel dysfunction (70.8%) and bladder dysfunction (62.0%)^29^. This relevant study, like ours, showed that physical health problems secondary to SCI, reported by participants, are extremely common worldwide and demand investment in appropriate management, medical care and preventative measures.

### Limitations

We recognize the limitations this online research and convenience sample. Unfortunately, in Brazil, we do not have an interconnected database, which allows us to have comparable statistical data. We also do not have specific government statistical data on the prevalence and incidence of SCI in the country. Faced with this reality, as researchers, created our own database with volunteers. Attentive to this demand, we found this online research alternative to get a sense, even if limited, to ensure that the patient’s opinion is considered in future scientific studies and in the development of clinical trials in Brazil.

Some international studies show that elderly people with chronic diseases do not always demonstrate readiness to use digital means to participate in research^31–33^. In contrast, the participants of this study, people suffering from SCI, are mostly young and at a productive age, which supports the suitability of the online data collection method adopted. In any event, the age and clinical situation of the patient should be considered when proposing research methods requiring digital devices (cellular phones, computers, etc.) and the ability and willingness to use virtual media. Another peculiarity of this study was the inclusion of a question on the digital form during data collection, which made it impossible for all the participants to answer the question.

## Conclusion

This study was ground-breaking in Brazil for considering the active participation of people with SCI and enabling the same to offer opinions on future topics of scientific research. The results obtained indicate the desire of people with SCI for investigations involving cellular regeneration (stem cells), rehabilitation, and cure. In contrast, the difficulties after SCI reported by the participants indicate a demand for studies that promote accessibility and locomotion, and assist in neurogenic bowel and bladder management, in sexuality, and in the return to work. In other words, the people with SCI priorities accessibility but wish to have some research about recovery and stem cell therapy.

This survey will aid rehabilitation professionals and clinical research organizations to engage stakeholders to implement a comprehensive, new and realistic national management program for the population. The results obtained empower people with SCI by enabling them to participate in the future design of scientific investigations, taking into consideration the desires and problems indicated by the participants themselves. Given the findings of this study, public and private investment in research on the participatory development style and rehabilitation of people with SCI in Brazil is essential, especially for the development of experimental studies, as their development depends on substantial financial investment.
